# Supplementary material for: Dose optimization of TBI-223 for enhanced therapeutic benefit compared to linezolid in antituberculosis regimen
Source: Nat Commun. 2024 Aug 25;15:7311. doi: 10.1038/s41467-024-50781-4 (PMC11344811; doi:10.1038/s41467-024-50781-4)
Supplement: Supplementary file 1 — Supporting Information [file 41467_2024_50781_MOESM1_ESM.pdf]

Supporting information

Figure S1: TBI-223 Phase 1 data, model and visual predictive check

Figure S2: Structural models

Figure S3: PK-PD VPC of linezolid in monotherapy studies

Figure S4: PK-PD VPC of TBI-223 in monotherapy studies

Figure S5: PK-PD VPC of linezolid in combination therapy studies

Figure S6: PK-PD VPC of TBI-223 in combination therapy studies

Figure S7: Linezolid shows a narrow therapeutic window and required frequent dose adjustments

Figure S8: Concentration-time profiles of TBI-223 in tuberculosis lesions.

Figure S9: Exposure ranges of mouse experiments and predicted human simulations

Table S1: Linezolid PK-PD parameters from different mouse infection models

Appendix A: Translational modeling of TBI-223 PK

Appendix B: Clinical simulation code

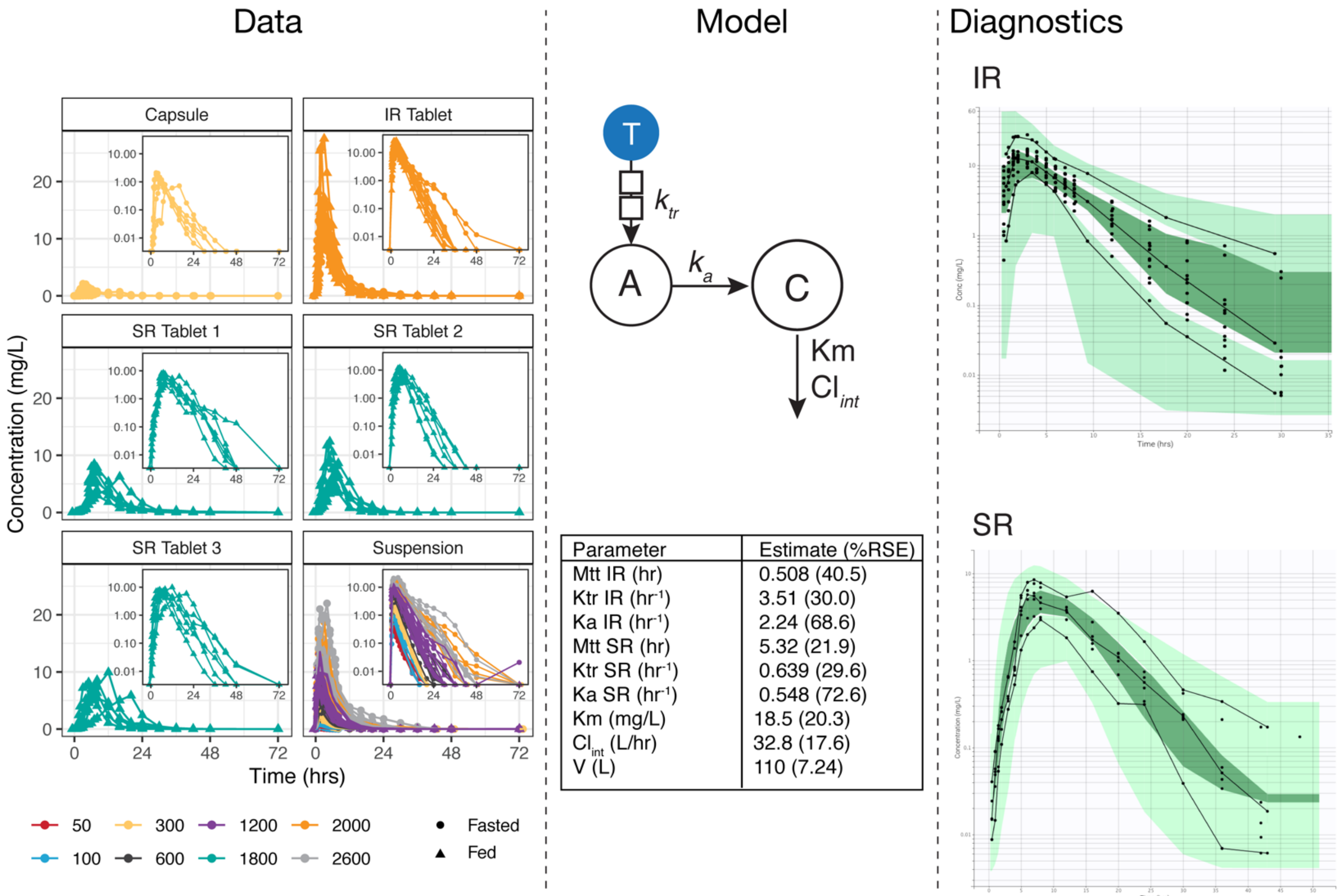

Parameter	Estimate (%RSE)
Mtt IR (hr)	0.508 (40.5)
Ktr IR (hr^{-1})	3.51 (30.0)
Ka IR (hr^{-1})	2.24 (68.6)
Mtt SR (hr)	5.32 (21.9)
Ktr SR (hr^{-1})	0.639 (29.6)
Ka SR (hr^{-1})	0.548 (72.6)
Km (mg/L)	18.5 (20.3)
Cl_{int} (L/hr)	32.8 (17.6)
V (L)	110 (7.24)

Figure S1: TBI-223 Phase 1 data, model and visual predictive check.

Concentration values after single dose administration of TBI-223 for 6 formulation types. Phase 1 patients received different doses in mg, as denoted by color, and were either fasted or fed, as denoted by data point shape. Concentration values are presented on a linear scale with inserts showing the same data log-scaled.

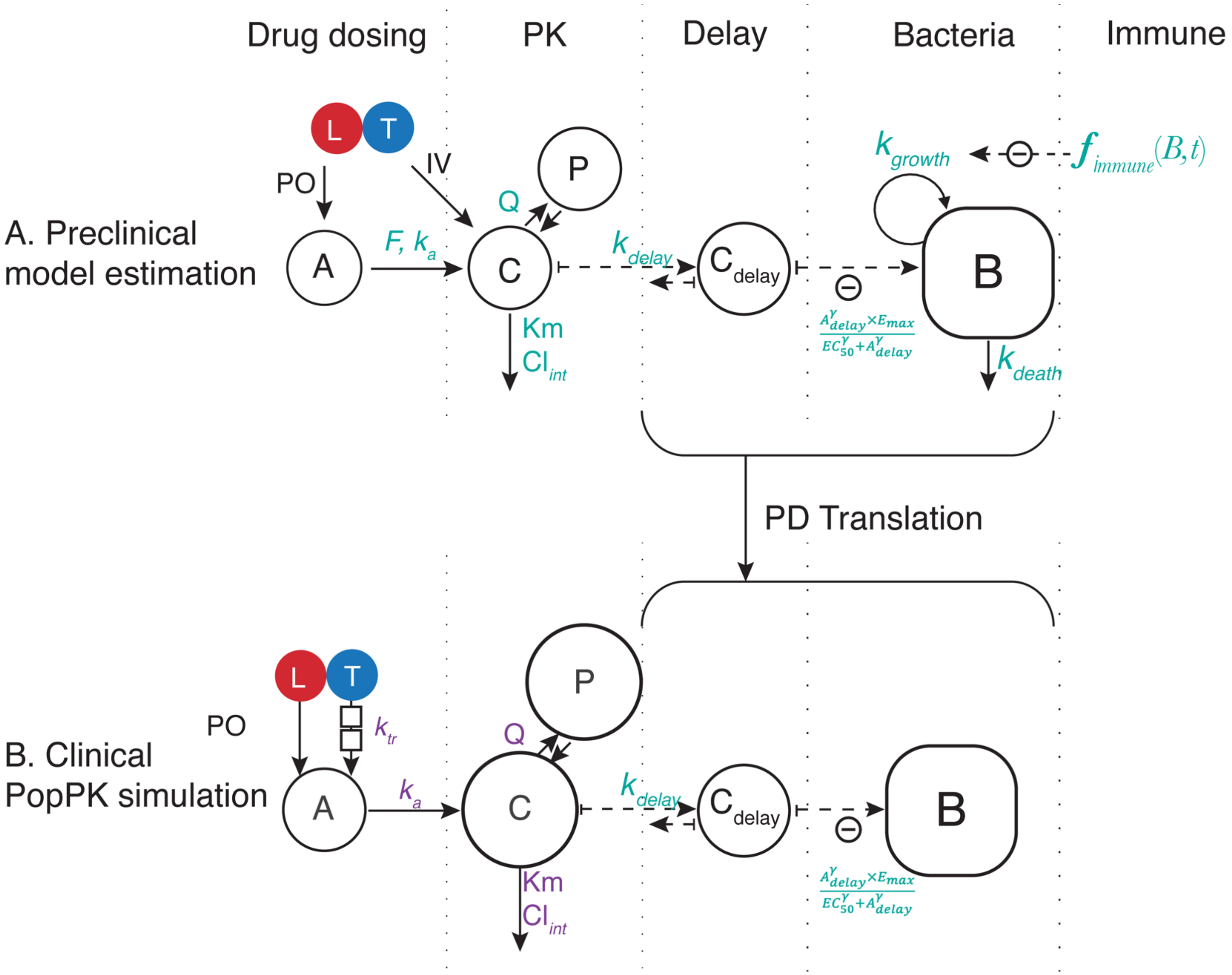

Figure S2: PK-PD structural model of translation.

Nonclinical data is used to estimate parameters in green in A. Clinical simulations then use parameters (purple) from a clinical population PK model (PopPK) to simulate concentrations that are input into a bacterial model using the nonclinical estimates from the previous step. L: Linezolid, T: TBI-223, PO: Per os (oral administration), IV: intravenous administration, compartment A: concentration in absorption compartment, compartment C: concentration in central (plasma measured) compartment, compartment P: concentration in peripheral compartment, compartment C_{delay}: concentration in delay compartment, compartment B: bacterial compartment, k_{tr}: rate of transit, k_a: rate of absorption, Q: intercompartmental clearance, Km: Michaelis-Menten Constant, Cl_{int}: Intrinsic clearance, k_{delay}: rate of delay

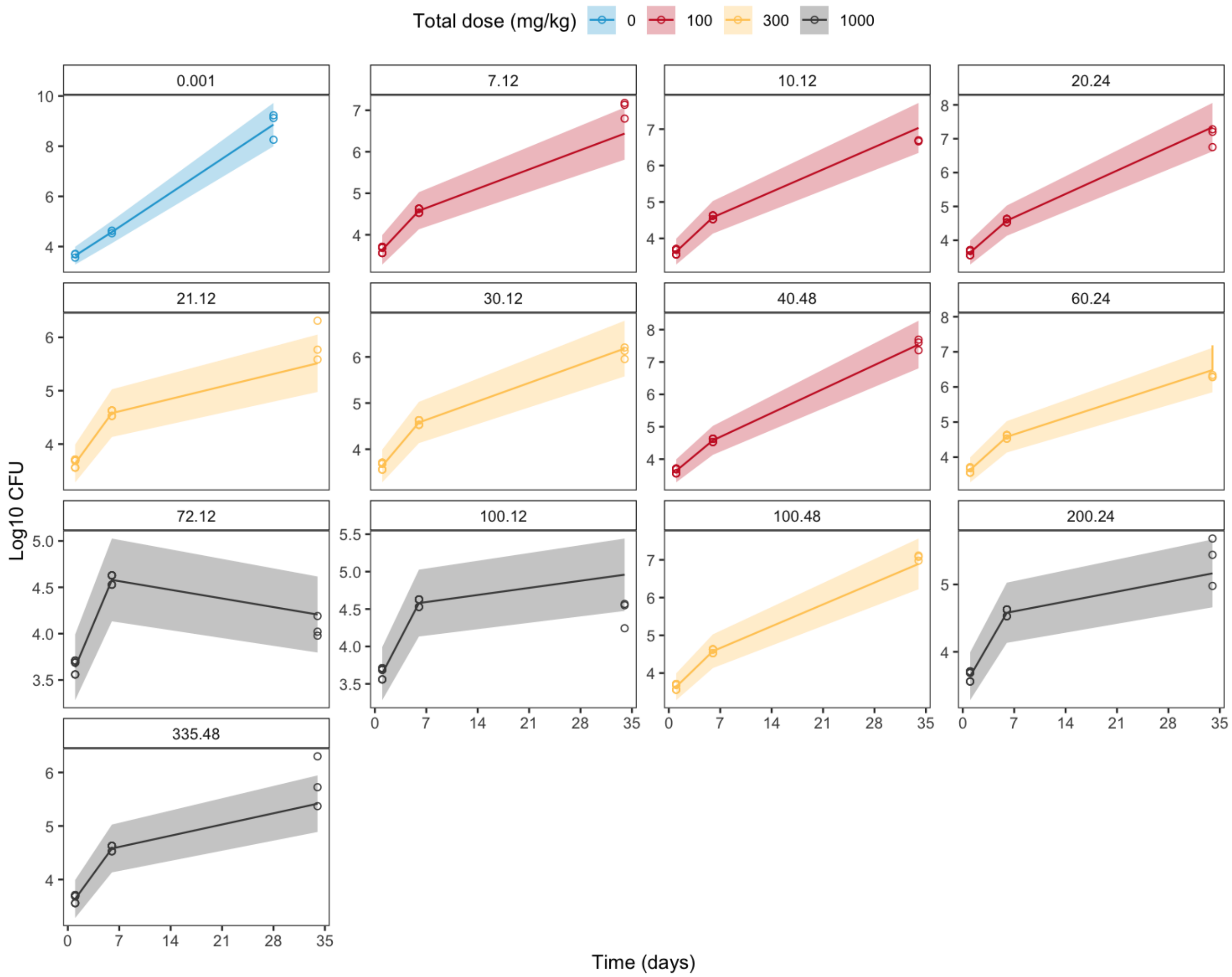

Figure S3: PK-PD visual predictive check of linezolid in monotherapy studies

The total weekly dose is denoted by varying colors. Each panel represents a distinct dosage schedule with observed data points marked as open circles. The model predicted 95th percentile interval is illustrated by the shaded area, with the median represented by a solid line.

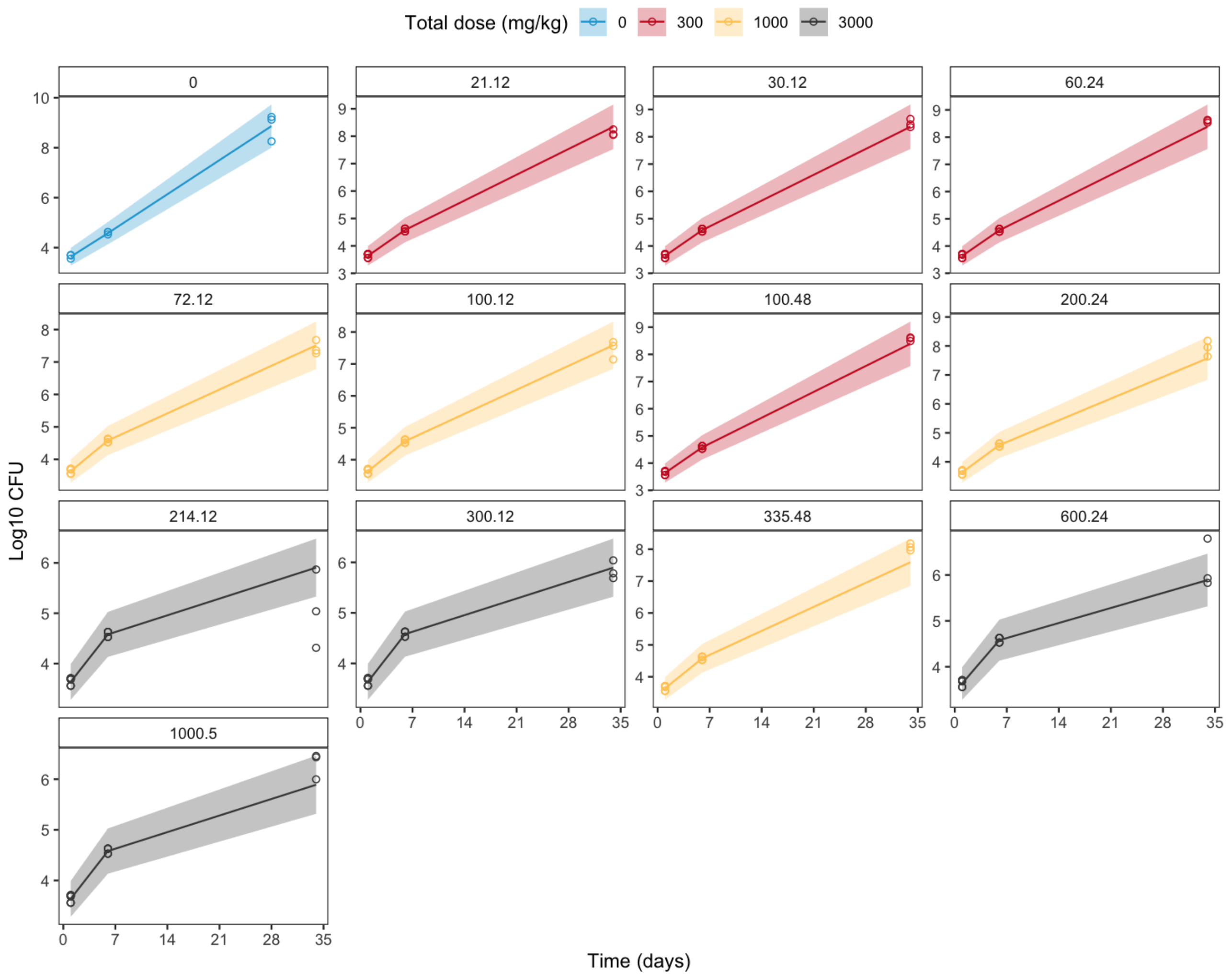

Figure S4: PK-PD visual predictive check of TBI-223 in monotherapy studies

The total weekly dose is denoted by varying colors. Each panel represents a distinct dosage schedule with observed data points marked as open circles. The model predicted 95th percentile interval is illustrated by the shaded area, with the median represented by a solid line.

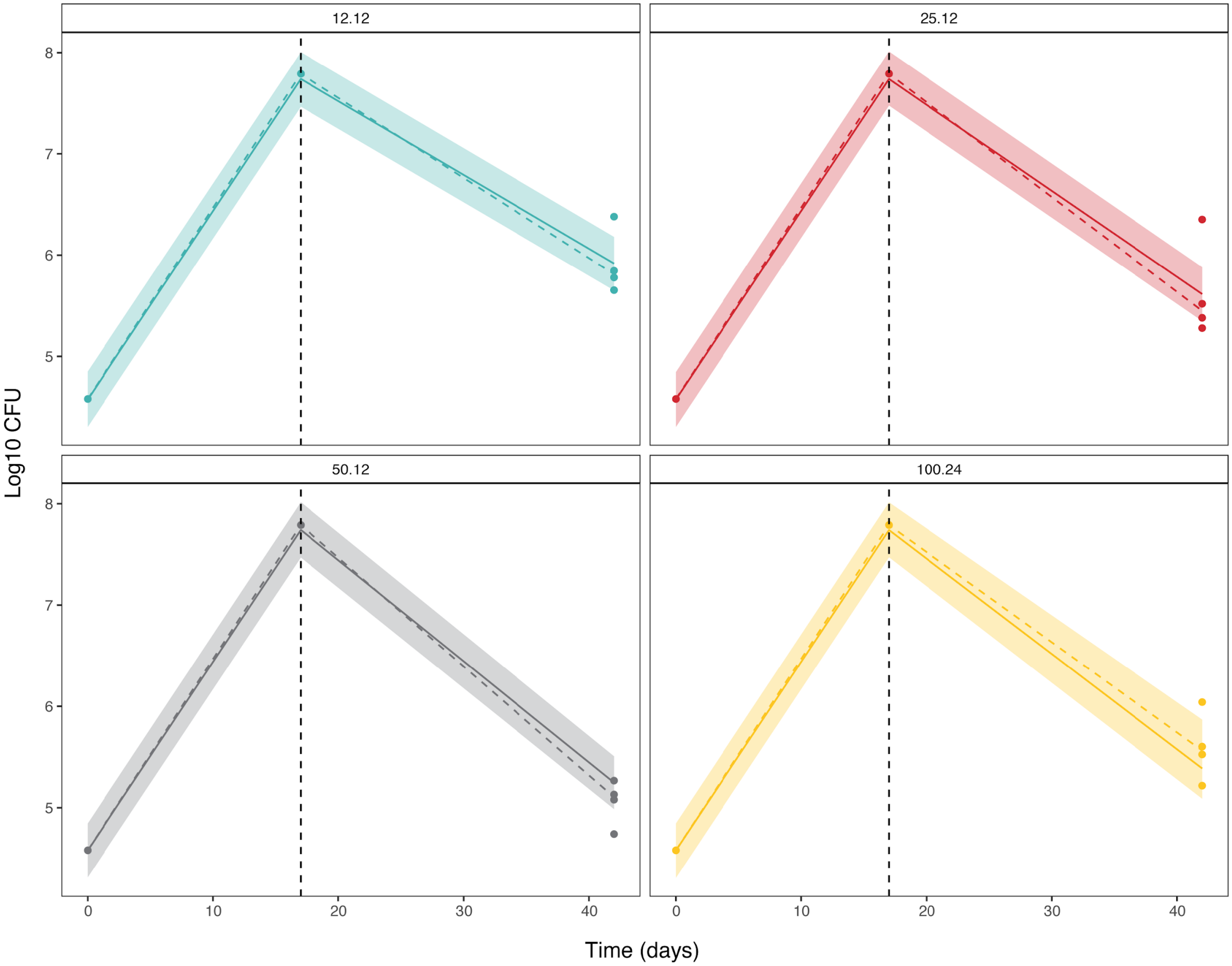

Figure S5: PK-PD VPC of linezolid in combination therapy studies

Each panel represents a distinct dosage schedule with observed data points marked as dots and the time of infection shown as a dashed line. The model predicted 95th percentile interval is illustrated by the shaded area, with the median represented by a solid line.

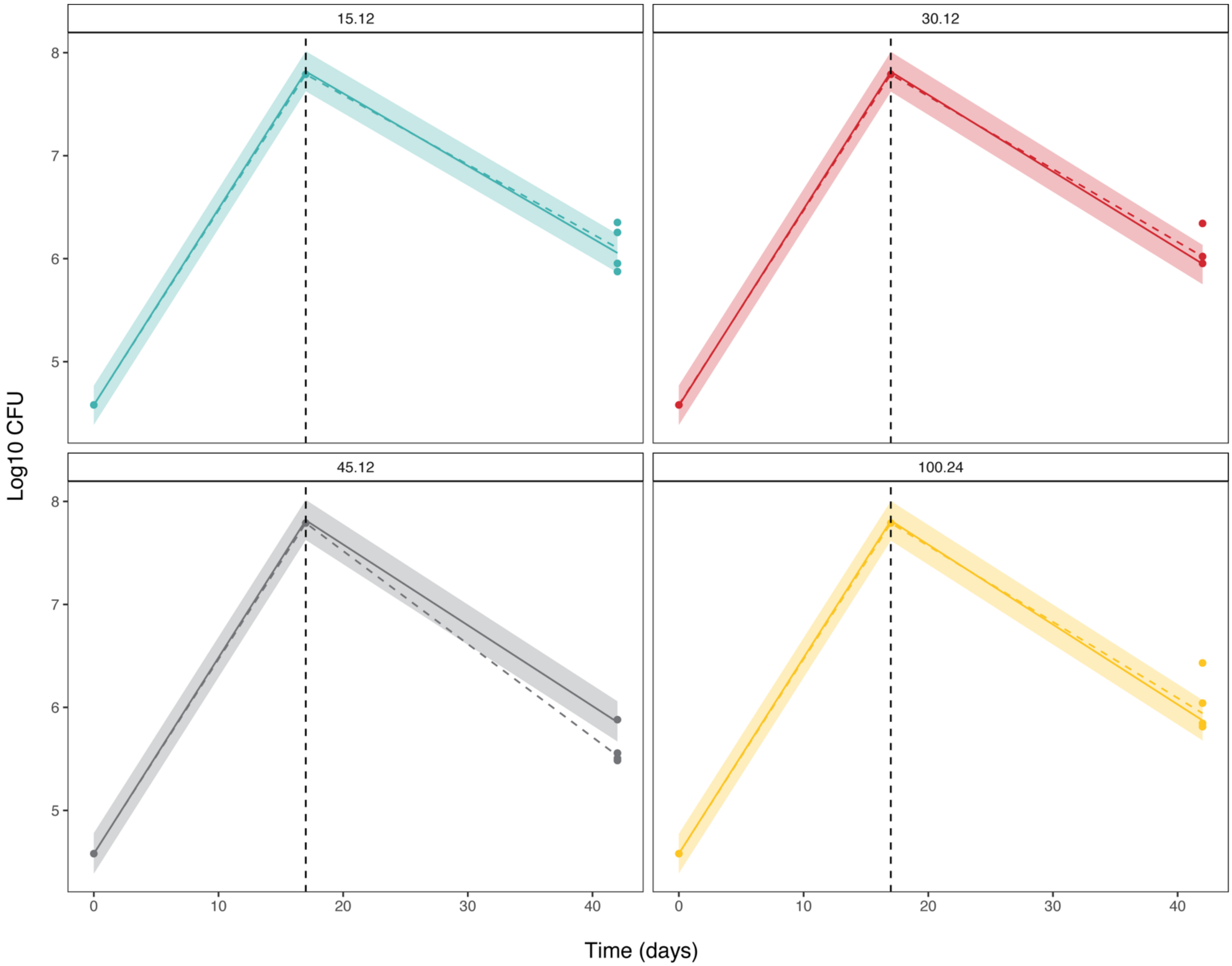

Figure S6: PK-PD VPC of TBI-223 in combination therapy studies

Each panel represents a distinct dosage schedule with observed data points marked as dots and the time of infection shown as a dashed line. The model predicted 95th percentile interval is illustrated by the shaded area, with the median represented by a solid line.

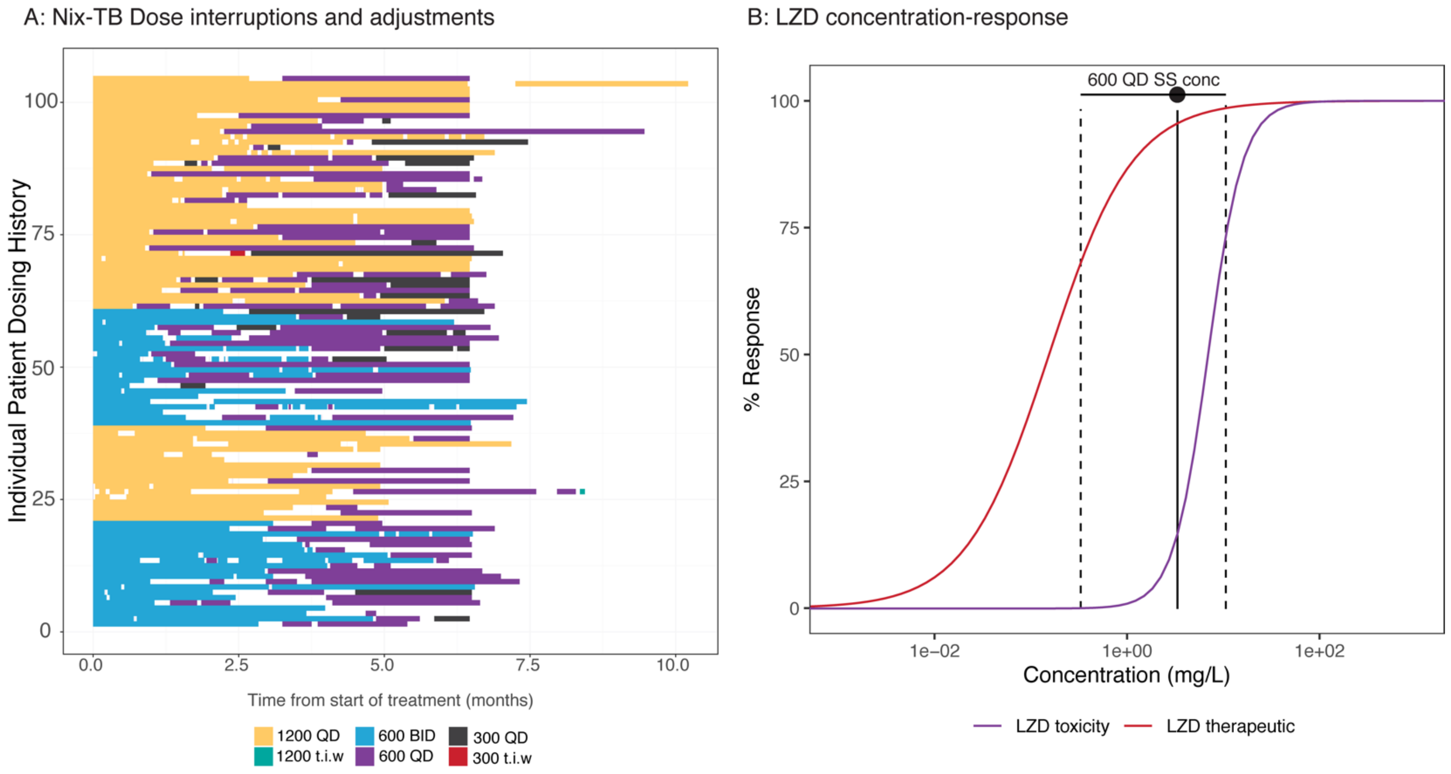

Figure S7: Linezolid shows a narrow therapeutic window and required frequent dose adjustments.

A. Individual linezolid dosing history for each patient in Nix-TB trial. White spaces indicate treatment interruption. Other colors indicate linezolid dose. B. Concentration-response of linezolid in combination with bedaquiline and pretomanid (blue) as determined by a sigmoidal PK-PD model from nonclinical data and the concentration-response of linezolid using a published red blood cell toxicity model (purple).

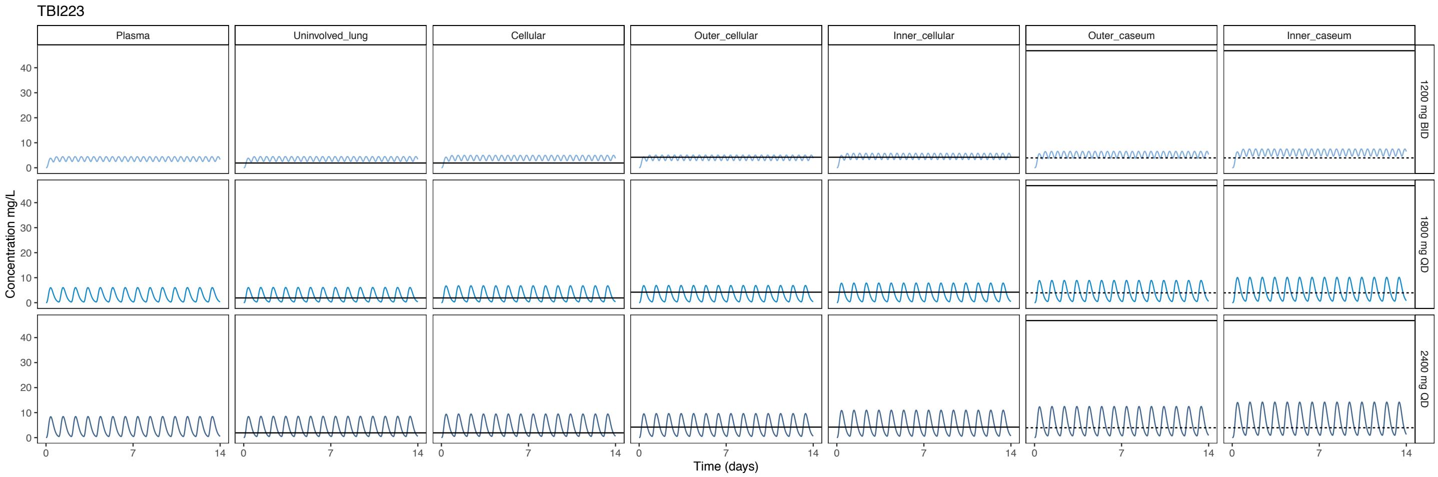

Figure S8: Concentration-time profiles of TBI-223 in tuberculosis lesions.

Median simulations of TBI-223 SR formulation in 7 tuberculosis lesions at 1200 mg BID, 1800 mg QD and 2400 mg QD. Solid lines represent lesion-specific target concentration for TBI-223 monotherapy and the dashed line represents combination therapy with bedaquiline and pretomanid. Target concentration for uninvolved lung is MIC (2 mg/L), for cellular lesions is macrophage IC_{90} (4.2 mg/L), and for caseous lesions is caseum MBC_{90} (46.8 mg/L for monotherapy TBI-223 and 3.9 mg/L for TBI-223 combined with equimolar amounts of bedaquiline and pretomanid).

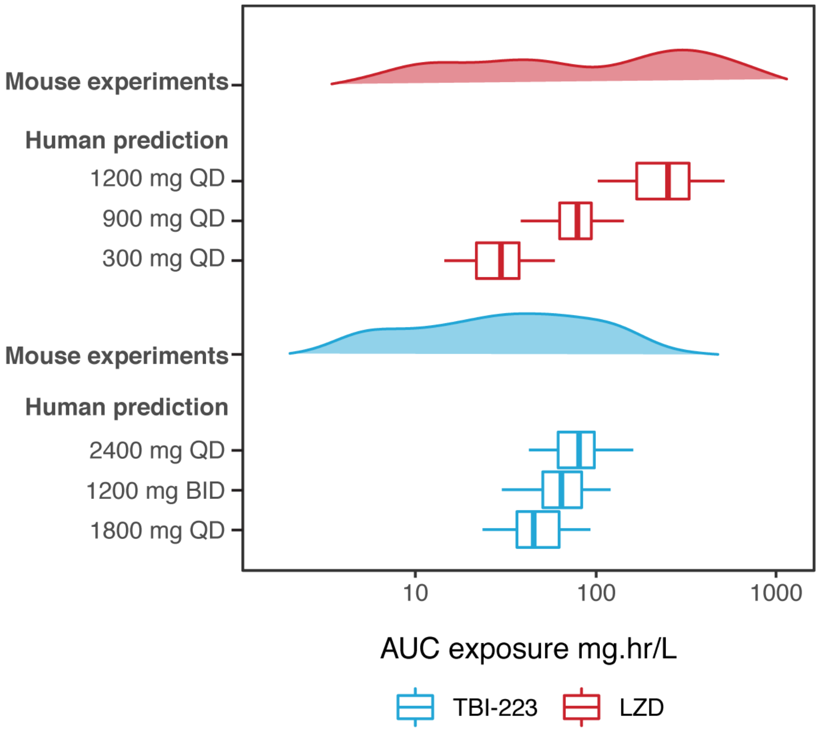

Figure S9: Exposure ranges of mouse experiments and predicted human simulations.

Distribution curves represent pharmacokinetic area under the curve over one week of dosing for all linezolid (LZD; red) and TBI-223 (blue) doses tested in the mouse efficacy model. Boxplots represent simulated human pharmacokinetic exposures over one week, with the median, 25th-75th percentiles, and whiskers indicating the 95% prediction interval. Distribution curves show the area under the curve for all doses tested in mouse efficacy models.

Table S1: Monotherapy linezolid PK-PD parameters from different mouse infection models

Parameter	Acute mouse model (6-day incubation)	Chronic mouse model (32-day incubation)
EC_{50} (mg/L)	2.87 (9.72)	4.56 (0.563)
Emax (day^{-1})	0.999 (2.30)	0.354 (17.9)
Rate of delay (day^{-1})	6.44 (0.01)	240 FIX

Appendix A: Translational modeling of TBI-223 PK

TB ALLIANCE
GLOBAL ALLIANCE FOR TB DRUG DEVELOPMENT

TB Alliance Study Number: TBI-223-NCLN-057
UCSF Study Number: UCSF TBI-223 Report 1 (Non-GLP)

UCSF Report Title: Translational modelling of linezolid and a new oxazolidinone antibiotic, TBI-223

Sponsor: Global Alliance for TB Drug Development
40 Wall Street 24th floor
New York, NY 10005
United States of America

Testing Facility: University of California San Francisco (UCSF)
Department of Bioengineering and Therapeutic Sciences
Byers Hall, 600 16th Street
UCSF Mission Bay Campus
San Francisco, CA, 94158
United States of America

Author:
Natasha Strydom, PhD
Position
University of California San Francisco
San Francisco, CA, 94158
United States of America

Date: October 23, 2018

Reviewed and Approved By:
Rada Savic, PhD
Associate Professor
University of California San Francisco
San Francisco, CA, 94158
United States of America

Date: October 23, 2018

Translational modelling of linezolid and a new oxazolidinone antibiotic, TBI-223

Natasha Strydom
Rada Savic

Date: 23 October 2018

Table of Contents

Translational modelling of linezolid and a new oxazolidinone antibiotic, TBI-223 2

1 Summary 4

2 Introduction 5

3 Methods 5

4 Results and discussion 6

- **4.1 Translational data** 6
- **4.2 Non-linear model** 6
- **4.3 Scaling** 7
- **4.4 Clinical extrapolation** 9

5 Conclusion 9

6 Figures 11

7 Tables 23

8 Supplementary 27

1 Summary

1. Linezolid (LZD) and TBI-223 (also known as OTB-223) have non-linear elimination and required a model-based approach using non-linear mixed effects to accurately estimate their pharmacokinetic parameters and extrapolate to human drug pharmacokinetics.

2. Using a non-linear model, extrapolated human LZD pharmacokinetics from preclinical *in vivo* experiments compared well with clinical parameters and simulated exposures, showing that the oxazolidinone class of antibiotics can benefit from non-linear modelling and allometric scaling to accurately predict human pharmacokinetics.

3. Using a similar model-based approach as used for LZD, the final model of TBI-223 was used to simulate human pharmacokinetic parameters and pharmacokinetic profiles at different dose levels. For a 100 mg, daily oral dose simulated to steady state, the non-linear model predicted a Cmax of 1.51 mg/L and AUC of 5.50 mg.hr/L.

2 Introduction

Pharmacokinetic profiles of linezolid (LZD) and TBI-223 revealed non-linear kinetics in pre-clinical (and clinical for LZD) studies suggesting saturation of the elimination pathway at higher concentrations. Pre-clinical to clinical scaling using non-compartmental analysis of drugs that show non-linear elimination and saturated absorption is likely to under-predict true drug pharmacokinetics as the elimination pathway of the drug becomes saturated leading to higher area under the curve (AUC) values and higher maximum concentrations. LZD is cleared approximately 65% by non-renal clearance and has two major metabolite pathways. *In vitro* studies have shown that linezolid metabolism is mediated by cytochrome P450 but is not a major elimination route and not fully described. *In vitro* TBI-223 rodent and non-rodent experiments showed different cytochrome profiles. To more reliably predict the pharmacokinetic (PK) properties of TBI-223 (and LZD) for human dosing and predict human pharmacokinetics, a model-based approach using non-linear mixed effects was used to estimate the non-linear clearance of the two oxazolidinone class antibiotics. The estimated PK parameters were then allometrically scaled by weight across 3 species to extrapolate to human PK parameters and more accurately simulate human exposures.

3 Methods

Concentration-time data for each drug in plasma were modeled using a population PK methodology, which is the appropriate method for separation of the signal from the noise. Data were fit to nonlinear mixed-effects models with first-order conditional estimation methods as implemented in the software Monolix (version 2018R1; Lixoft). Simulations were performed with open-source software R (version 3.3.1) using the mlxR package from Lixoft.

The likelihood ratio test was used to evaluate statistical significance for inclusion of additional parameters in nested models, assuming the objective function value (OFV), which is proportional to -2 times log likelihood, is chi-squared distributed; thus, a decrease in OFV of 3.84 points among models with one parameter differing is considered a statistical difference with 5% significance level.

Model building to best scale animal data proceeded with model building for each animal separately and starting with intravenous (IV) data to first establish the structural model and estimate initial clearance and volume parameters. From here individual animal specific parameters were compared to observe scaling trends. The animal data was then combined with clearance (Cl) and volume (V) scaled by weight according to initial relationships observed. The scaling factors were fixed to 0.75 and 1 and later freed to ensure best scaling.

4 Results and discussion

4.1 Translational data

PK data was collected for LZD and TBI-223 in mice, rats, and dogs. Experiments included single oral and single bolus IV administration. The concentration-time profiles of the data collected and used for modelling are presented in Figure 1. In total, there were 238 number of observations for LZD, which included 53 in dogs, 45 in rats, and 140 in mice, and 330 number of observations for TBI-223, which included 141 in C57BL/6 mice, 45 in BALB/c mice, and 144 in dogs. The cytochrome profiles of TBI-223 were not included with the assumption that like LZD, the cytochrome pathway may not be the be a major metabolism pathway.

4.2 Non-linear model

As seen in the log-scaled oral profiles of LZD and TBI-223 in Figure 1, the rate of decrease in drugs at higher concentration are slower and increase when the concentration is lower indicating non-linear clearance. To account for the saturation of the elimination pathway at higher concentrations, Michaelis-Menten kinetics on elimination (example for IV bolus administration shown in equation 1) was necessary to fit the data, using parameters Vmax and Km. The maximum elimination rate (Vmax) was transformed to estimate *in vivo* clearance intrinsic (CL_{int}) in the model, Equation 2. This was important for scaling by weight across species as CL_{int} (L/hr) scaled better to weight than Vmax (mg/hr). For both drugs a two-compartment model with non-linear clearance fit best with the final structural model and corresponding equations used for both drugs are shown in Figure 2 and equations 3-5.

$$\frac{dA_c}{dt} = -\left(\frac{Vmax \times A_c}{V_c \cdot Km + A_c}\right)$$ **Equation 1**

$$Vmax = CL_{int} \times Km$$ **Equation 2**

$$\frac{dA_a}{dt} = -k_a \text{x} A_a$$ **Equation 3**

$$\frac{dA_c}{dt} = k_a \text{x} A_a - \left(\frac{Vmax \times A_c}{V_c \cdot Km + A_c}\right) - \frac{Q}{V_c} \times A_c + \frac{Q}{V_p} \times A_p$$ **Equation 4**

$$\frac{dA_p}{dt} = \frac{Q}{V_c} \times A_c - \frac{Q}{V_p} \times A_p$$ **Equation 5**

The compartments Aa, Ac and Ap refer to the absorption (used for oral doses), central and peripheral compartments. Km (mg/L) is half the concentration when Vmax is reached.

4.3 Scaling

To combine the animal models and scale allometrically across species, weight was introduced as a covariate on CL_{int}, inter-compartmental clearance (Q) and both central and peripheral volumes, example of individual parameter scaling shown in equation 6. Power scaling represented by β was applied to the log transformed individual weight (Wt_i) centered around median weight ($\widetilde{Wt}$) and initially set to ¾ on clearances and 1 for the volumes. Later the power scaling parameters were freed to best estimate the true allometric scaling of the drugs studied.

$$\bar{V}_i = V_{pop}\left(\frac{Wt_i}{\widetilde{Wt}}\right)^{\beta_V} \quad \text{and} \quad \bar{CL}_i = CL_{pop}\left(\frac{Wt_i}{\widetilde{Wt}}\right)^{\beta_{CL}}$$ **Equation 6**

TBI-223 showed traditional ¾ power scaling on CL_{int} and Q, with linear scaling on central (Vc) and peripheral volumes (Vp) with no notable change in likelihood (Delta OFV= 4.41 for 2 estimated exponents) if exponents were fixed or estimated. The LZD observations best fit a ⅔ power model on CL_{int}, ½ on Q and 0.9 for both volumes (Table 1) with notable change in likelihood (Delta OFV= 23.1 for 3 estimated exponents).

TBI-223 modelling revealed a dose effect on bioavailability (F) effect where increased dosing decreased F unrelated to the change in exposure due to non-linear clearance, Figure 3. Possibly this dose effect could be due to saturated absorption of TBI-223 at higher doses or limitations of the formulation used during dosing. Dose was included as a covariate in the model and F estimated categorically for each dose. Estimated bioavailability for each dose is presented in Table 1A and ranged from 9.6% for the 500 mg/kg dose and 62.8% for the 25 mg/kg dose relative to the intravenous dose.

The final model visual predictive checks and PK parameters of LZD and TBI-223 are shown in Figure 4 and Table 1A and 1B. Additional model diagnostics of covariate results, observations vs

individual predictions and individual fits can be found in the Supplementary section under Figures S1-S4.

4.4 Clinical extrapolation

Using the allometrically-scaled PK parameters of each drug, clinical PK parameters were extrapolated to a 60kg human using the same covariate relationship described in equation 6 (Figure 5). Inter-individual variability was set to 30% and bioavailability of TBI-223 fixed to 100% reference from the animal intravenous dose, to simulate highest possible exposure and ensure maximum concentrations were within safety range. We have also examined the scenario where oral F was set to the 25 mg/kg dose (1500 mg in a 60kg human) bioavailability of 62.8% to compare the range of exposure with decreased absorption amount. Absorption rate which differs greatly from the fast absorbing animal gavage dosing to human dosing was fixed to 1.34 to simulate absorption over a 30-minute period.

Simulated exposure levels from extrapolated LZD parameters when compared to its known clinical exposures compared well, Table 2 and Figure 6, indicating that the model-based method performed well for the clinically used compound. Additional simulations for LZD and TBI-223 were used to evaluate PK profiles and exposure over a range of doses, Table 3 and 4 and Figures 7 and 8, respectively.

5 Conclusion

A non-linear model based on Michaelis-Menten saturated kinetics was successfully used to fit the preclinical data of TBI-223 and LZD. This model was allometrically scaled between species and human PK parameters extrapolated from preclinical results. The assumption was made that the Michaelis-Menten constant is unchanged between species, while other pharmacokinetic parameters follow allometric scaling principles. Simulated outcomes of LZD compared well to

known clinical exposure, emphasizing the benefit of non-linear mixed effects modelling for the oxazolidinone antibiotic. TBI-223 simulations were performed at a range of suggested first in human doses to observe predicted exposure. Assuming 100% bioavailability, at an initial oral dose of 100 mg, it is predicted that maximum concentrations will be below 2 mg/L. Potential dose-dependent decrease in bioavailability observed in animals could appear in humans and show lower exposures than predicted. Additional simulations at 63% were performed and predicted that maximum concentration will be below 1.7 mg/L, with an approximate 45% decrease in AUC.

6 Figures

A: TBI-223 preclinical data

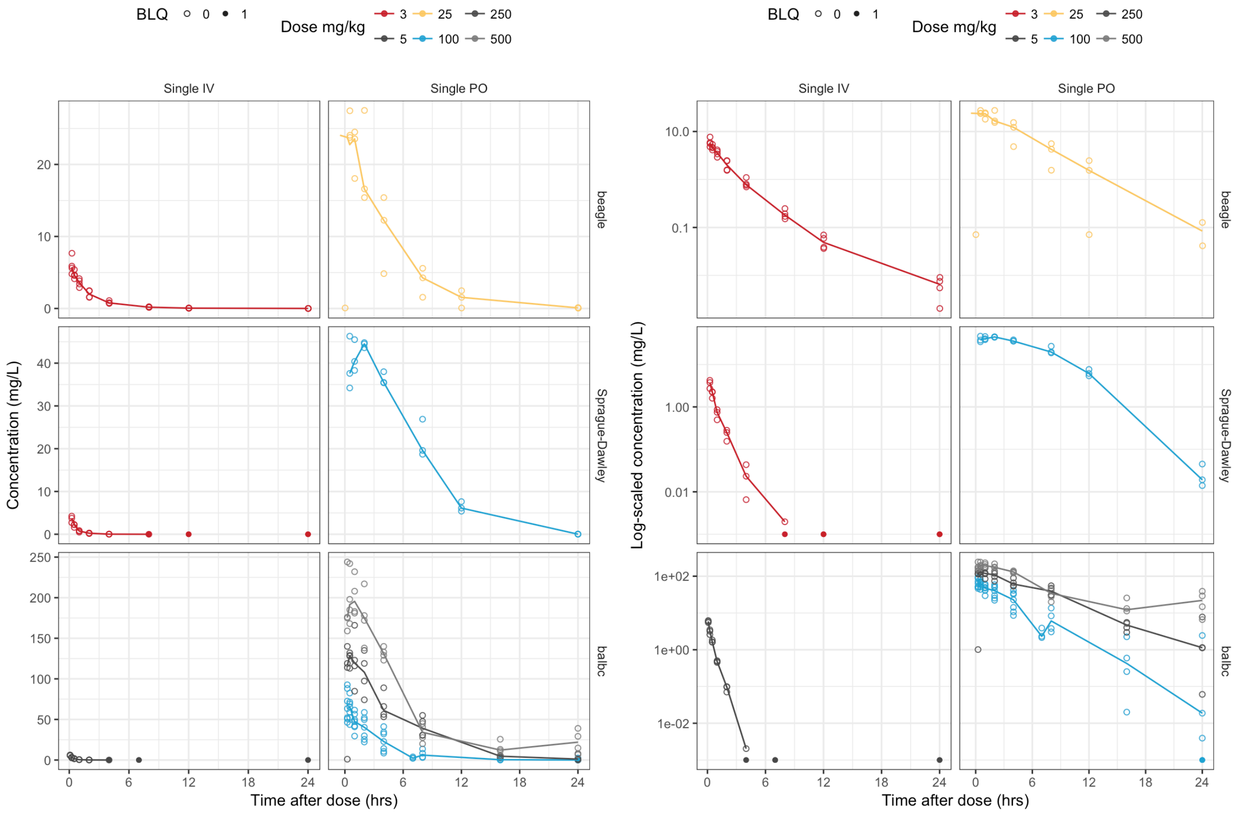

B: LZD Preclinical Data

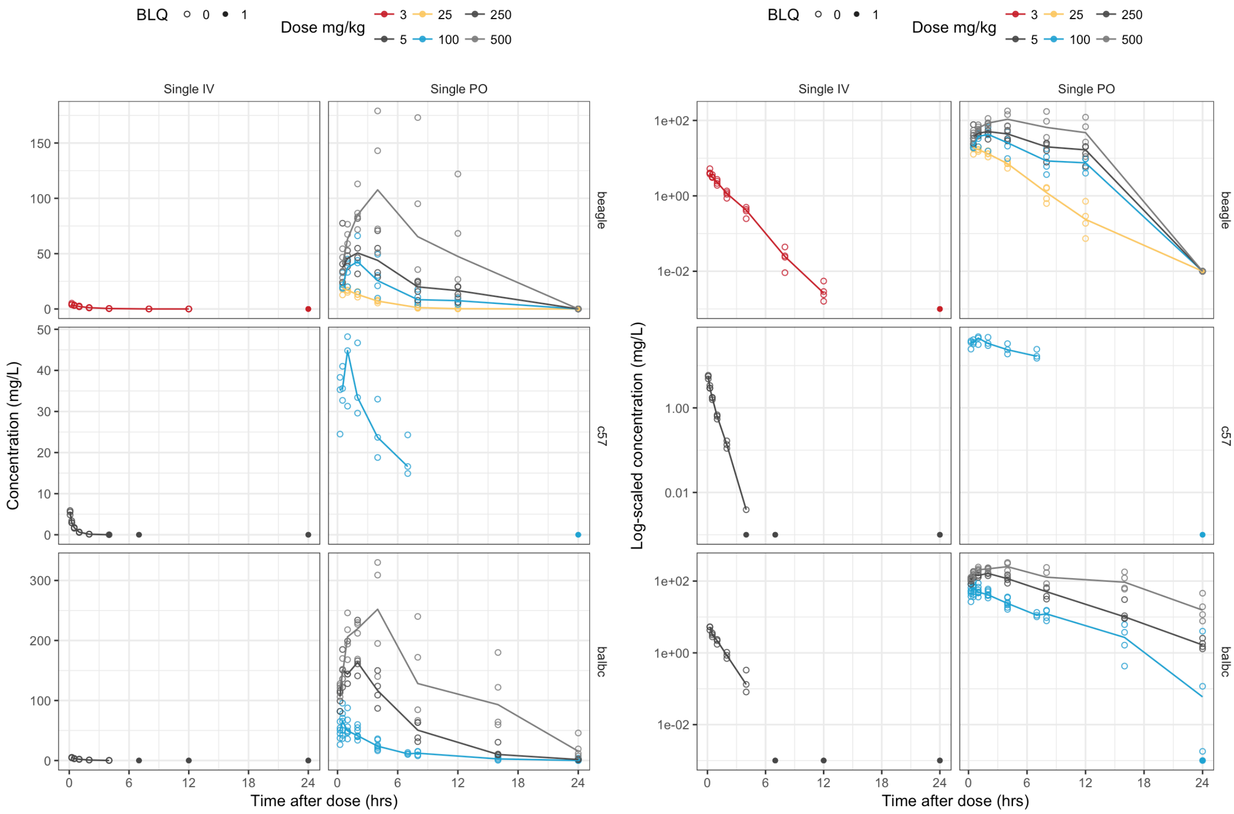

Figure 1: TBI-223 and LZD animal data

Animal data is shown by species (beagle dog (beagle), C57/BL6 mice (c57), BALB/c mice (balbc)) and dosage route. Concentration-time observations are represented by open circles and the lines representing the median of the observations grouped by dose (mg/kg). Below the limit of quantification (BLQ) data is shown in solid circles and were incorporated into the model using the M3 method.

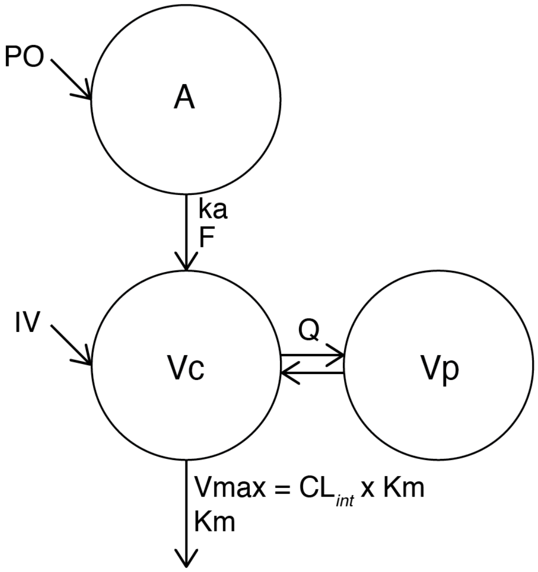

Figure 2: Schematic of structural model

A 2-compartment model with Michaelis-Menten elimination best fit the data. Intravenous data was coded to enter the central compartment and oral data the absorption compartment with absorption rate (ka) and fraction absorbed (F), i.e. bioavailability, estimated.

TBI-223 Bioavailability

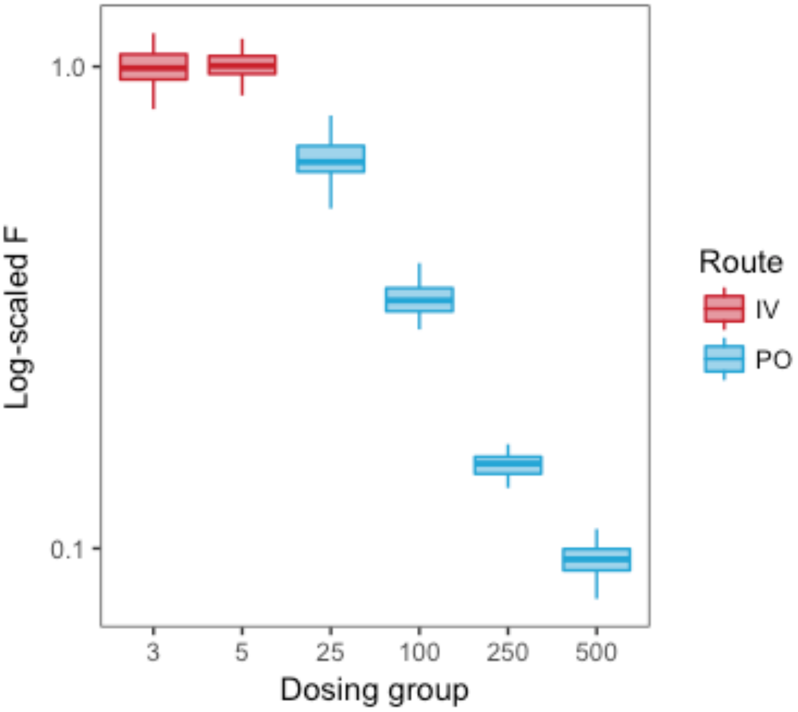

Figure 3: Bioavailability of TBI-223 vs dosing group

A decrease in individual predicted fraction of absorption (F), i.e. bioavailability, is observed with increasing dosing relative to IV. For this plot, individual F values were estimated with individual variability to capture the distribution of observed bioavailability. The final model had no individual variability on F as its exclusion had no significant impact on OFV.

LZD

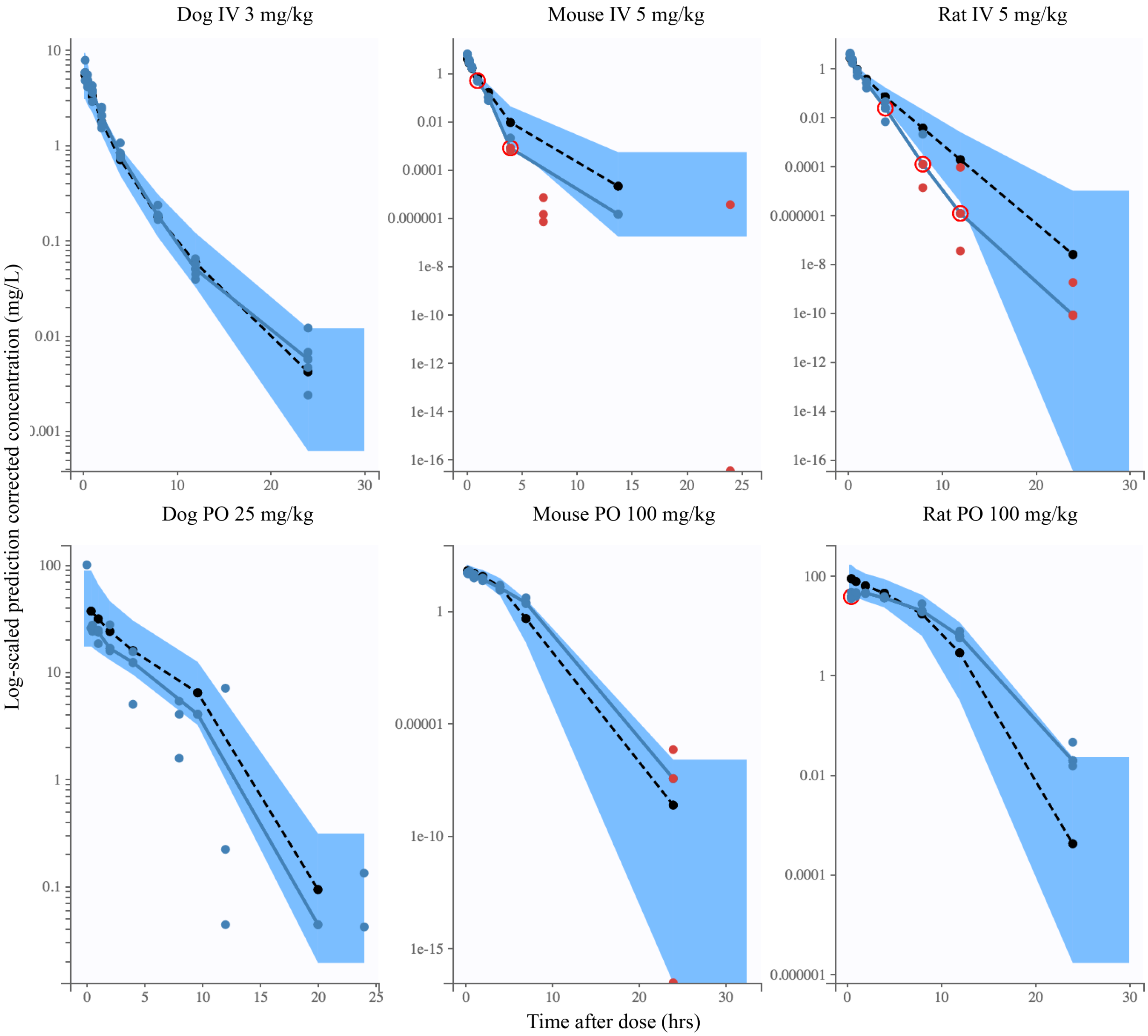
Dog IV 3 mg/kg
Mouse IV 5 mg/kg
Rat IV 5 mg/kg
Dog PO 25 mg/kg
Mouse PO 100 mg/kg
Rat PO 100 mg/kg
Log-scaled prediction corrected concentration (mg/L)
Time after dose (hrs)

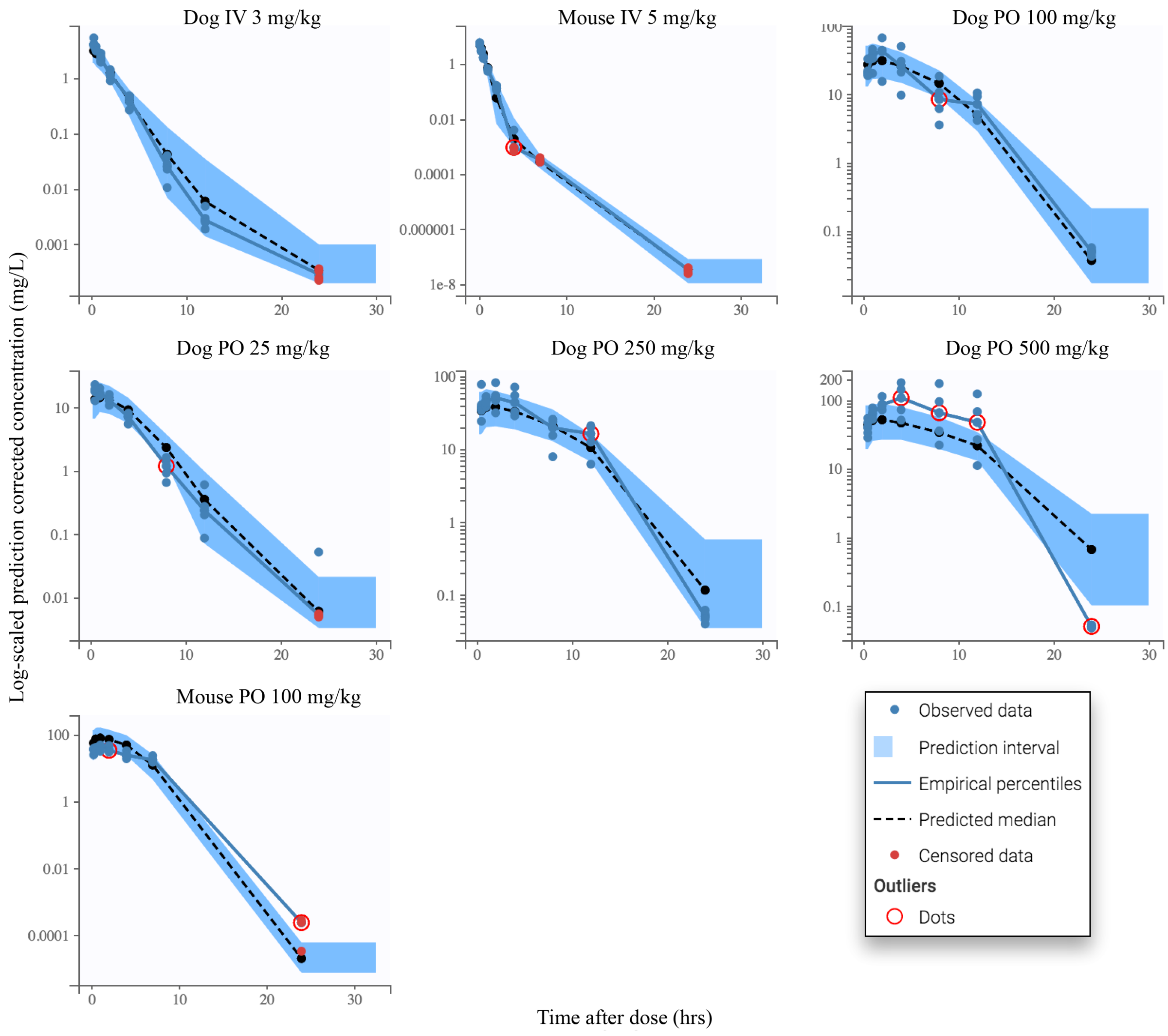

Figure 4: Visual predictive check of LZD and TBI-223

The visual predictive checks were simulated for 1000 animals using final inter-species model with allometric scaling. The median simulated concentration-time profiles are represented by the dashed black line to represent the median, with the shaded light blue area representing the 50% prediction interval of the 1000 subjects. The original observed data is overlaid on top of the simulated bands as blue circles and the median represented as a solid blue line to assess the

ability of the model to capture both the median and distribution of the preclinical data. The red circles represent the simulated observations that were below the limit of quantification (BLQ).

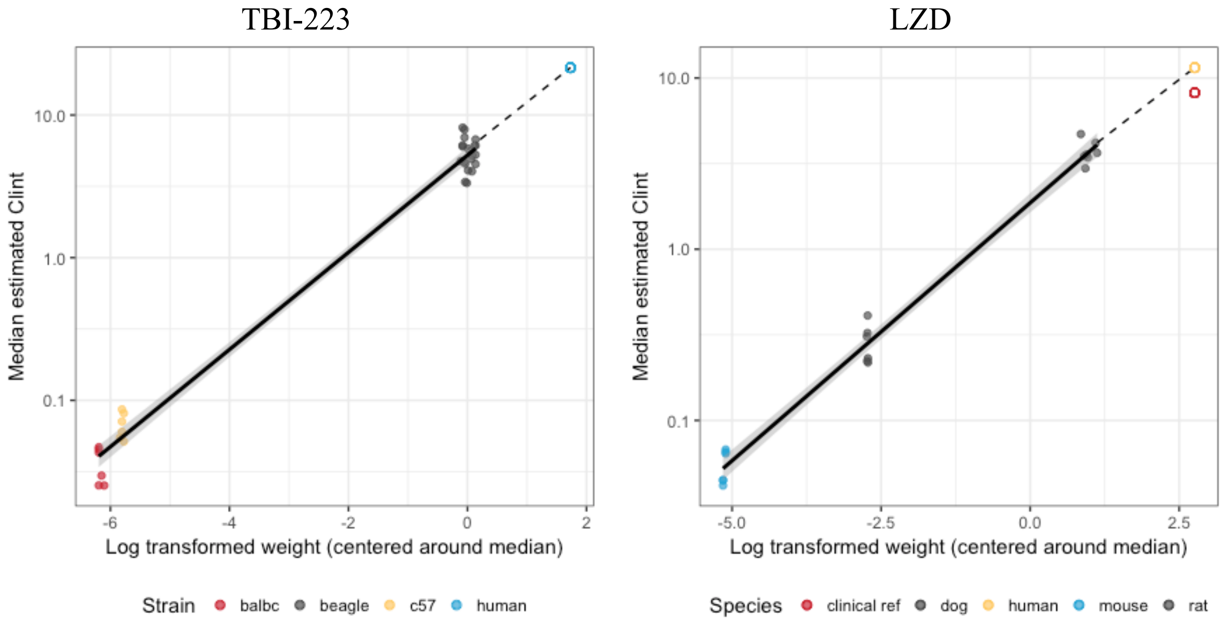

Figure 5: Allometrically scaled clearance across species

Example of allometric scaled parameter clearance to obtain human parameter estimates for A: TBI-223 and B: LZD. LZD includes reference clinical clearance estimated from the Nix-TB trial as red circle.

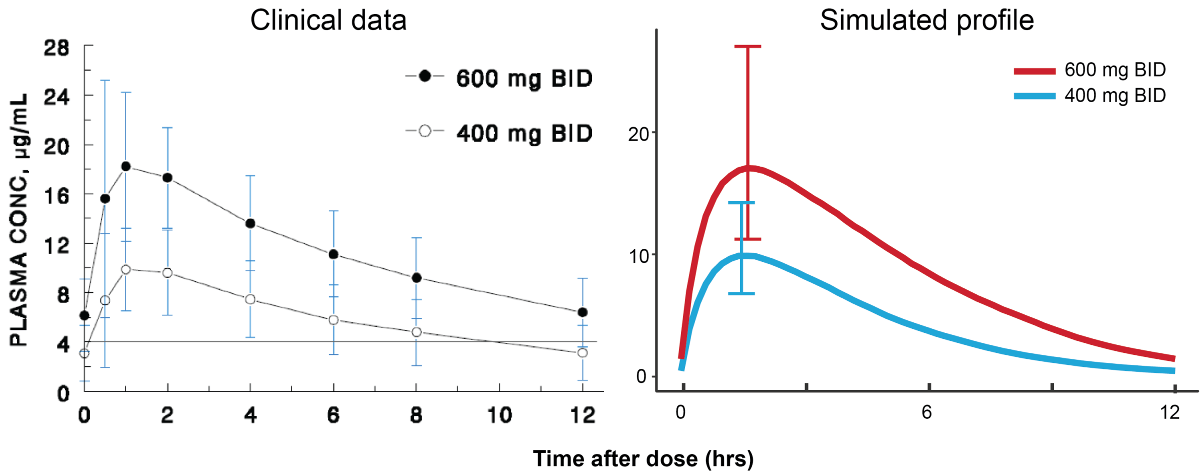

Figure 6: LZD PK profiles from clinical data and simulated data

The left panel shows clinically observed LZD pharmacokinetic profiles after a 600 mg and 400 mg twice daily dose at steady state (data from Pfizer ZYVOX package insert). Using the extrapolated model, the same doses were simulated to steady state and the 12 hrs profiles shown in the right panel, with 5 and 95 percentiles shown at C_{max}.

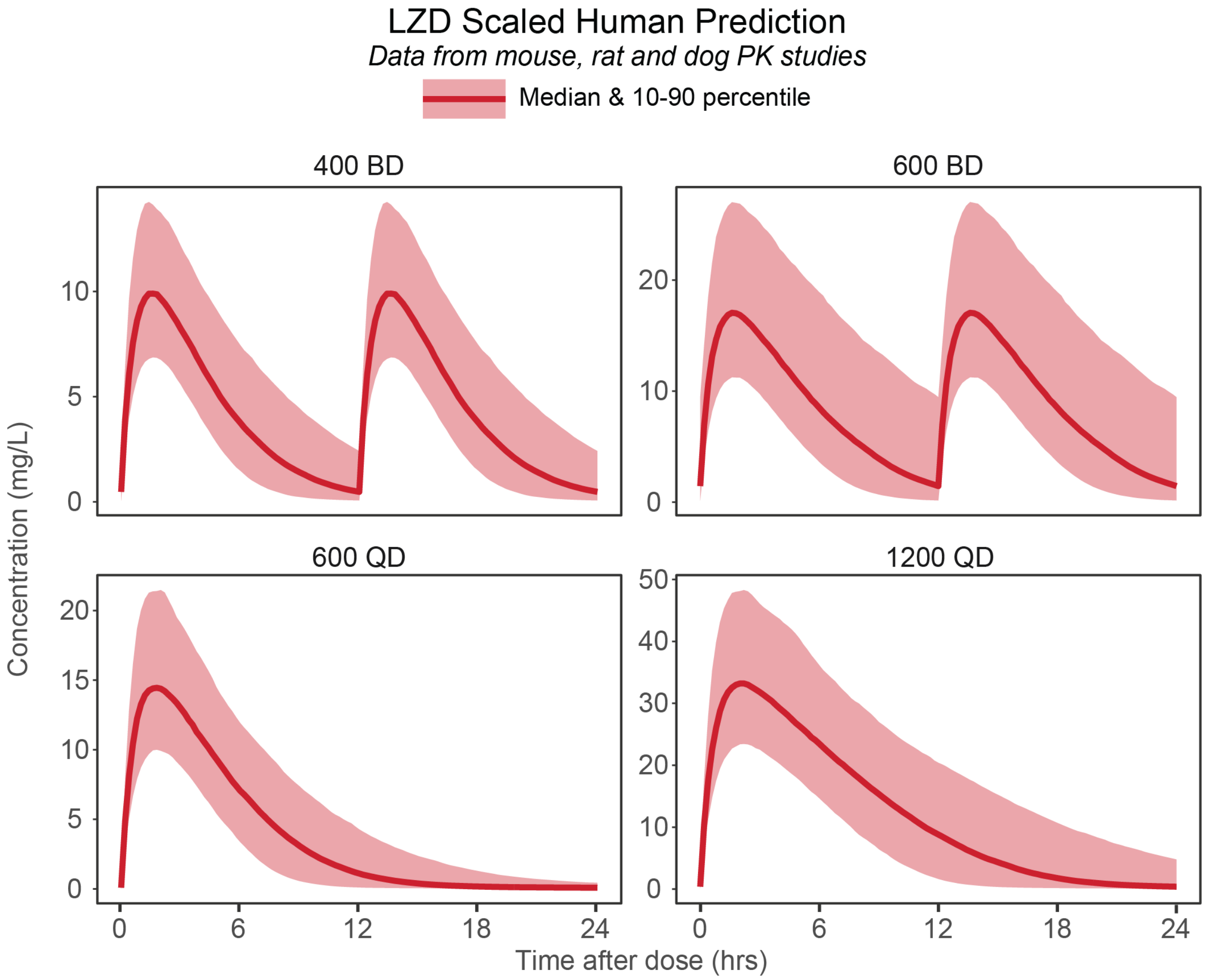

Figure 7: Simulated human LZD profiles

Pharmacokinetic concentrations-time profiles were simulated to steady state for 1000 subjects at different doses. The red line represents the median profile and the red shaded area the 10-90 percentile range of the simulated profiles.

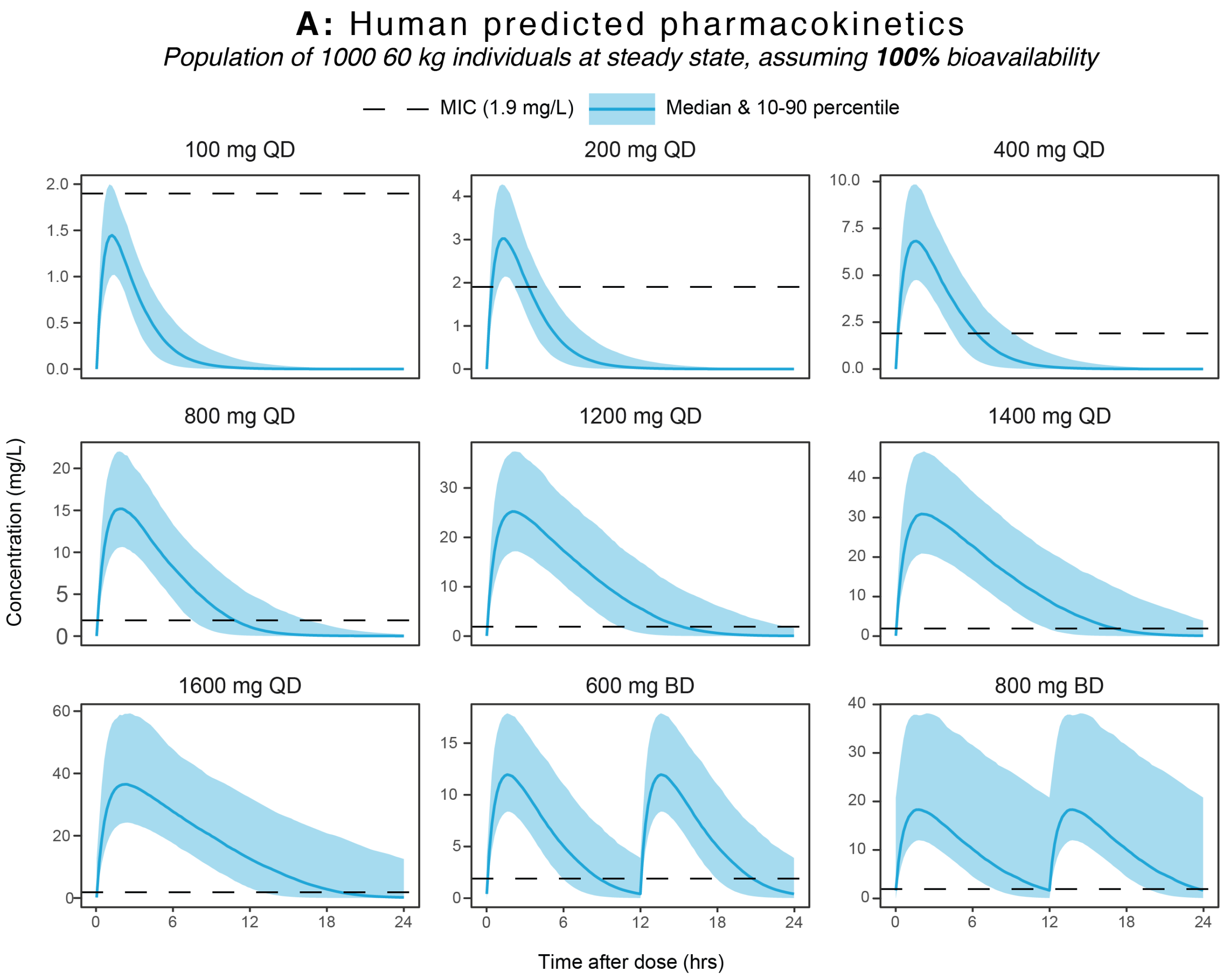

A: Human predicted pharmacokinetics
Population of 1000 60 kg individuals at steady state, assuming 100% bioavailability
MIC (1.9 mg/L)
Median & 10-90 percentile
100 mg QD
200 mg QD
400 mg QD
800 mg QD
1200 mg QD
1400 mg QD
1600 mg QD
600 mg BD
800 mg BD
Concentration (mg/L)
Time after dose (hrs)

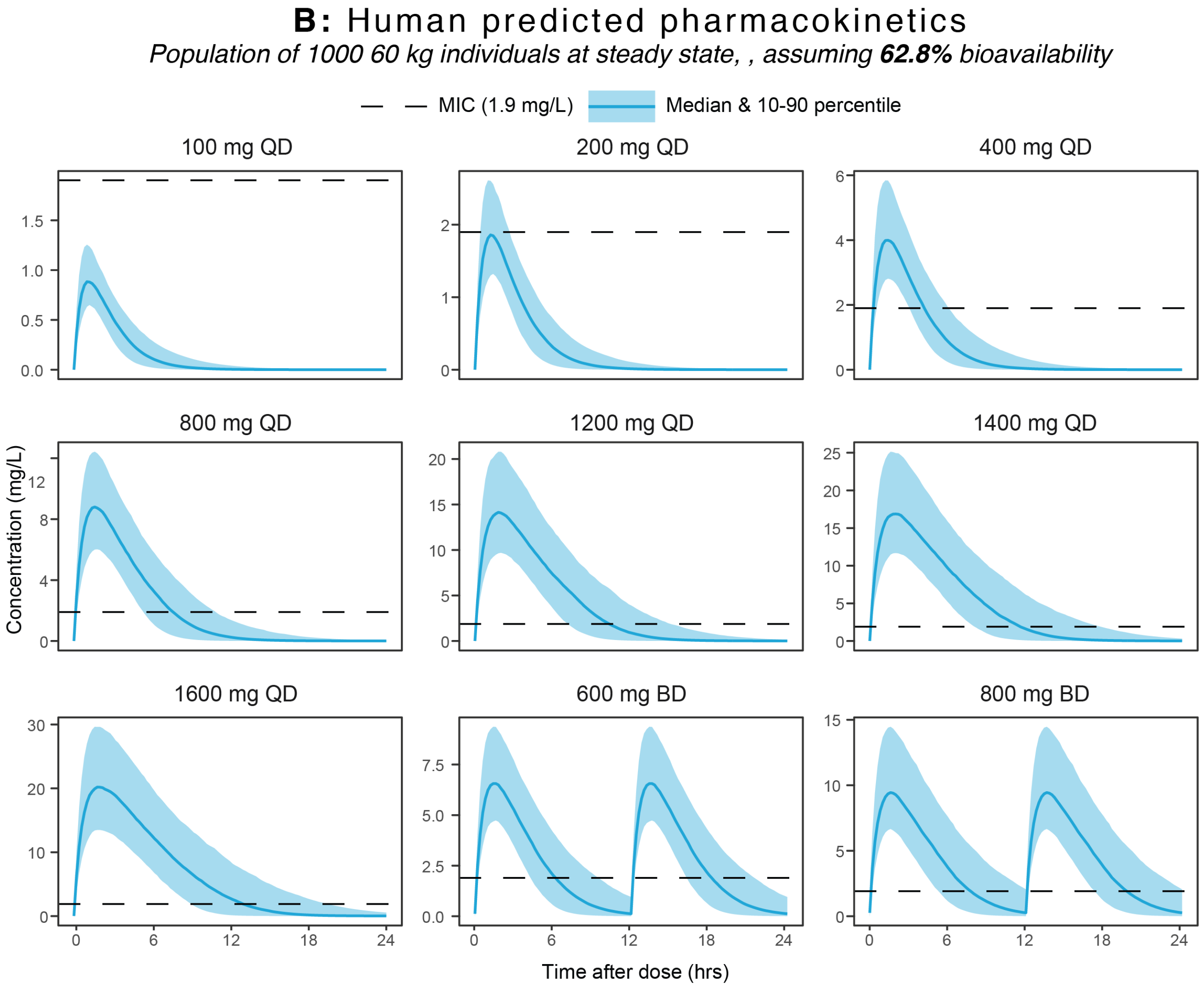

Figure 8: Simulated human TBI-233 profiles

Pharmacokinetic concentrations-time profiles were simulated to steady state for 1000 subjects at different doses, assuming A: 100% bioavailability and B: 62.8% bioavailability. The blue line represents the median profile and the blue shaded area the 10-90 percentile range of the simulated profiles. The dashed black line represents TBI-223's MIC of 1.9 mg/L.

7 Tables

Table 1A: TBI-223 Final parameters

TBI-223 Parameter	*ß-scaling factor*	Estimated animal parameters (RSE)	Projected human parameters
Median weight (kg)	-	10.3	60
CL_{int} (L/hr)	0.75	5.73 (33.8)	21.5
Km (mg/L)	-	5.32 (30.2)	5.32
Vc (L)	1.00	6.50 (5.91)	38.9
Vp (L)	1.00	0.20 (5.66)	1.17
Q (L/hr)	0.75	0.026 (2.85)	0.098
ka (hr^{-1})	-	3.9 (58.0)	1.34 FIX
F	-	-	IV Ref; 1.0<br>PO; 0.638
$F_{25\ mg/kg}$	-	0.638 (52.0)	-
$F_{100\ mg/kg}$	-	0.317(27.1)	-
$F_{250\ mg/kg}$	-	0.150 (12.6)	-
$F_{500\ mg/kg}$	-	0.0955 (5.60)	-

Table 1B: LZD Final parameters

LZD Parameter	*ß-scaling factor*	Estimated animal parameters (RSE)	Projected human parameters
Median weight (kg)	-	3.8	60
CL_{int} (L/hr)	0.67 (1.72)	1.74 (2.88)	11.05
Km (mg/L)	-	8.03	8.03
Vc (L)	0.9 (7.09)	2.00 (23.1)	38.9
Vp (L)	0.9 (13.6)	0.94 (9.02)	11.3
Q (L/hr)	0.5 (19.1)	0.06 (23.1)	0.23
ka (hr^{-1})	-	10 FIX	1.34 FIX
F	-	0.82 (8.31)	0.82

Table 2: LZD PK compared between the model and clinical exposure

	Clinical LIN-CL-001*	Predicted LZD
**600 mg QD**		
AUC_{0-24}(mg.h/L)	104	92.7 (59.4-146)
C_{max} (mg/L)	13.4	14.9 (10.5-21.0)
**600 mg BD**		
AUC_{0-24}(mg.h/L)	346.5	288 (126-483)
C_{max} (mg/L)	24.4	17.2 (11.9-29.0)
**1200 mg QD**		
AUC_{0-24}(mg.h/L)	300.4	292 (180-556)
C_{max} (mg/L)	30.8	34.5 (23.5-51.2)

*Clinical data from Pfizer ZYVOX package insert

Table 3: Predicted LZD pharmacokinetics of a median patient at steady state

Dosing	$AUC_{0\text{-}24}$(mg.h/L)	C_{max} (mg/L)	T_{max} (hr)	Time above MIC* (hrs)	Time above MIC* (%)
600 QD	92.7 (59.4-146)	14.9 (10.5-21.0)	1.2 (1.4-2.2)	14.2 (9.4-22.4)	59.2 (39.2-93.3)
400 BD	108 (65.7-188)	10.1 (7.03-14.6)	1.6 (1.2-2.2)	23.2 (15.2-24)	96.7 (63.3-100)
600 BD	288 (126-483)	17.2 (11.9-29.0)	1.8 (1.4-2.2)	24.0 (18.4-24)	100 (76.7-100)
1200 QD	292 (180-556)	34.5 (23.5-51.2)	2.2 (1.6-2.8)	22.6 (14.4-24)	91.7 (60.0-100)

*MIC = 0.5 mg/L

1000 patients simulated with assumptions that median absorption time takes 30 min and variance is 30% for CL_{int}, Vc, Vp, Q and ka.

Median values shown with 10 and 90 percentile range shown in parenthesis.

Table 4: Predicted TBI-223 pharmacokinetics of a median patient at steady state

Dosing	AUC_{0-24}(mg.h/L)	Tmax (hrs)	Cmax (mg/L)	Time above MIC* (hrs)	Time above MIC* (%)
Assuming **100%** relative bioavailability:					
50 QD	2.55 (1.74-3.75)	1.2 (0.8-1.6)	0.711 (0.516-0.980)	0.0 (0.0-0.0)	0
100 QD	5.54 (3.72-8.02)	1.2 (1.0-1.6)	1.43 (1.06-2.09)	0.0 (0.0-1.0)	0 (0-4.2)
200 QD	12.8 (8.46-19.7)	1.4 (1.0-1.8)	3.15 (2.19-4.47)	2.8 (1.6-4.4)	11.7 (6.7-18.3)
400 QD	33.3 (21.7-53.8)	1.6 (1.2-2.0)	6.91 (4.84-9.95)	6.2 (4.2-8.8)	25.8 (17.5-36.7)
800 QD	101 (61.7-168)	1.8 (1.4-2.4)	15.7 (10.6-22.7)	10.8 (7.6-15.8)	45.0 (31.7-65.8)
1200 QD	202 (121-344)	2.2 (1.6-2.8)	24.9 (17.0-36.9)	15.1 (10.0-22.8)	62.9 (41.6-95.0)
1400 QD	275 (168-513)	2.2 (1.6-3.0)	30.4 (20.1-47.0)	17.2 (11.2-24)	71.7 (46.7-100)
1600 QD	359 (211-876)	2.2 (1.8-3.0)	37.1 (24.7-60.8)	19.2 (13.0-24)	80.0 (54.2-100)
600 BD	133 (79.0-267)	1.4 (1.4-2.2)	12.2 (8.24-18.9)	17.6 (12.0-24)	73.3 (50.0-100)
800 BD	233 (128-699)	1.8 (1.6-2.2)	18.6 (11.7-37.2)	23.6 (14.8-24)	98.3 (61.7-100)
Assuming **63.8%** relative bioavailability:					
50 QD	1.55 (2.34-1.06)	1.2 (0.8-1.6)	0.445 (0.329-0.620)	0.0 (0.0-0.0)	0
100 QD	3.06 (2.03-4.68)	1.2 (1.0-1.6)	0.889 (0.635-1.27)	0.0 (0.0-0.0)	0
200 QD	6.90 (4.47-10.2)	1.4 (1.0-1.8)	1.86 (1.33-2.59)	1.0 (0.0-2.2)	4.17 (0-9.17)
400 QD	16.8 (10.7-25.7)	1.6 (1.2-2.0)	3.98 (2.79-5.70)	4.0 (2.6-5.8)	16.7 (10.8-24.1)
800 QD	46.1 (29.2-74.5)	1.8 (1.4-2.4)	8.79 (6.05-13.0)	7.5 (5.2-10.8)	31.3 (21.7-45.0)
1200 QD	89.6 (56.3-148)	2.0 (1.6-2.8)	14.7 (10.0-21.2)	10.2 (7.0-14.8)	42.5 (29.1-61.7)
1400 QD	116 (70.7-199)	2.2 (1.6-3.0)	17.3 (11.8-25.6)	11.4 (8.0-17.6)	47.5 (33.3-73.3)
1600 QD	147 (87.7-251)	2.4 (1.8-3.0)	20.3 (14.2-29.5)	12.8 (9.0-19.4)	53.3 (37.5-80.8)
600 BD	58.9 (37.4-98.2)	1.4 (1.4-2.2)	6.47 (4.50-9.35)	12.0 (8.0-18.8)	50.0 (33.3-78.3)
800 BD	95.1 (57.7-168)	1.8 (1.6-2.2)	9.33 (6.42-14.0)	15.2 (10.4-24)	63.3 (43.3-100)

*MIC = 1.9 mg/L

1000 patients simulated with assumptions that median absorption time takes 30 min and variance is 30% for CL_{int}, Vc, Vp, Q and ka.

Median values shown with 10 and 90 percentile range shown in parenthesis.

8 Supplementary

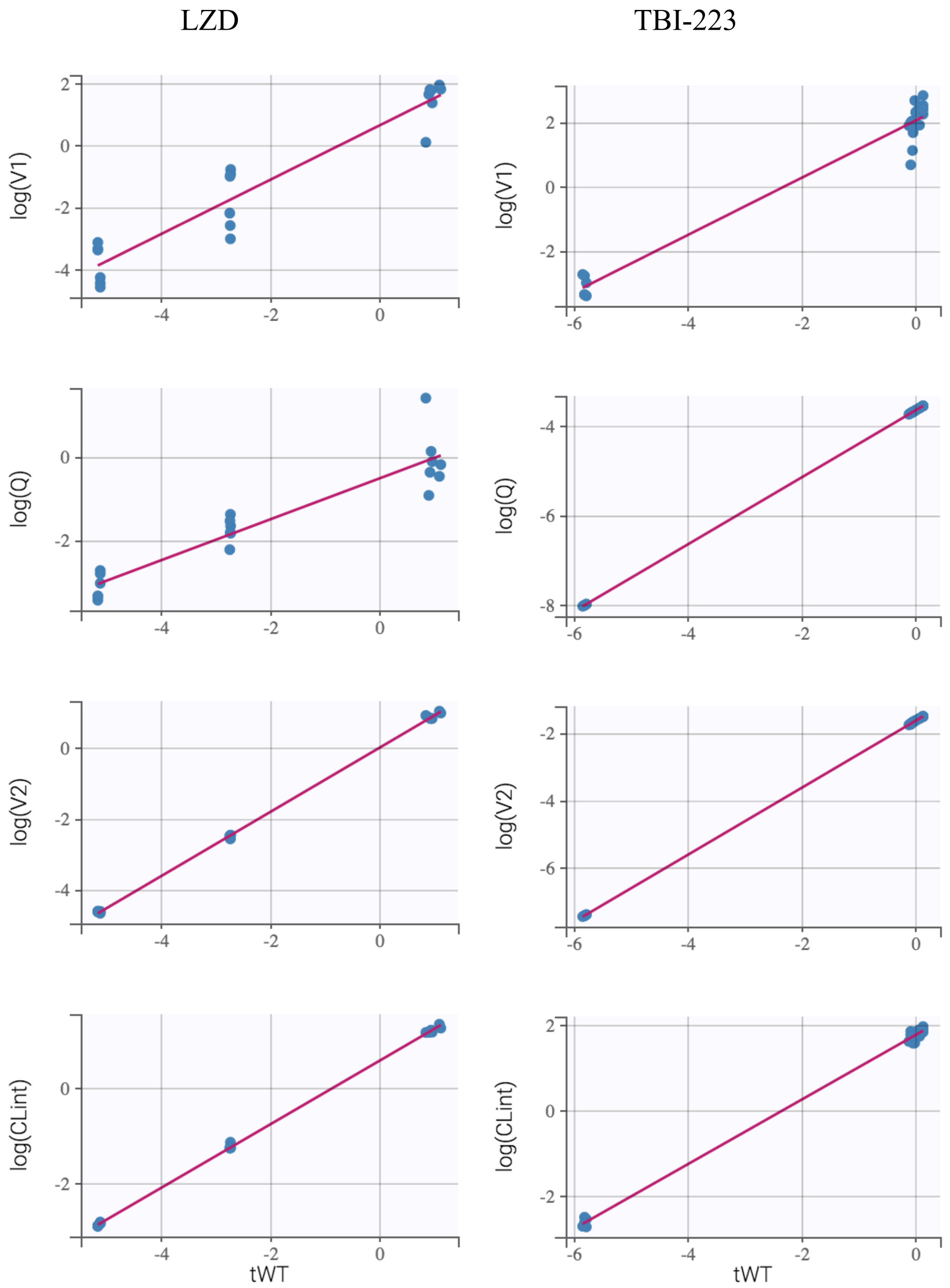

Figure S1: Estimating β scaling factor across species during model development for LZD and TBI-223

LZD

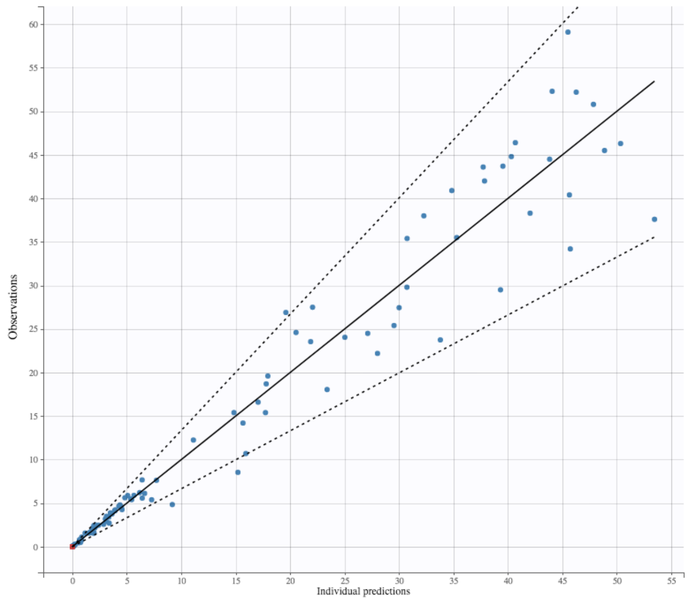

TBI-223

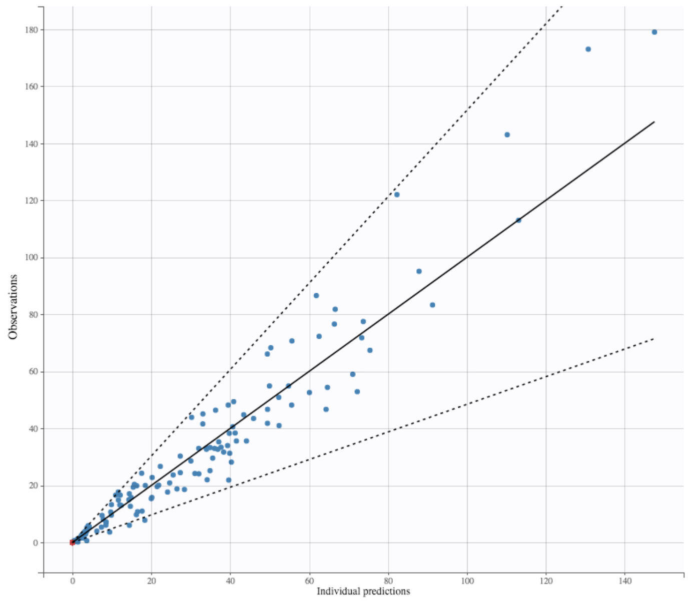

Figure S2: Model diagnostic check of observations vs individual predictions for LZD and TBI-223

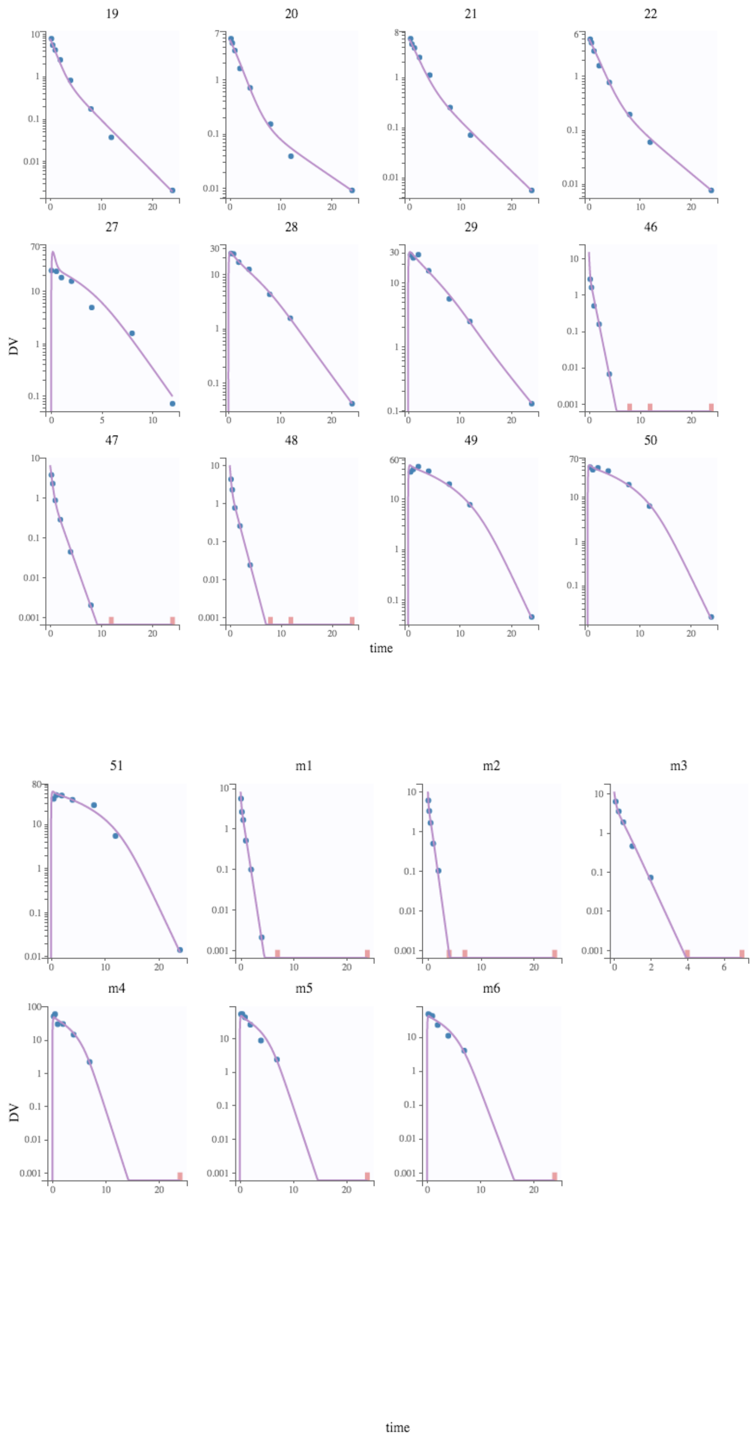

Figure S3: Model diagnostic check of individual fits of LZD

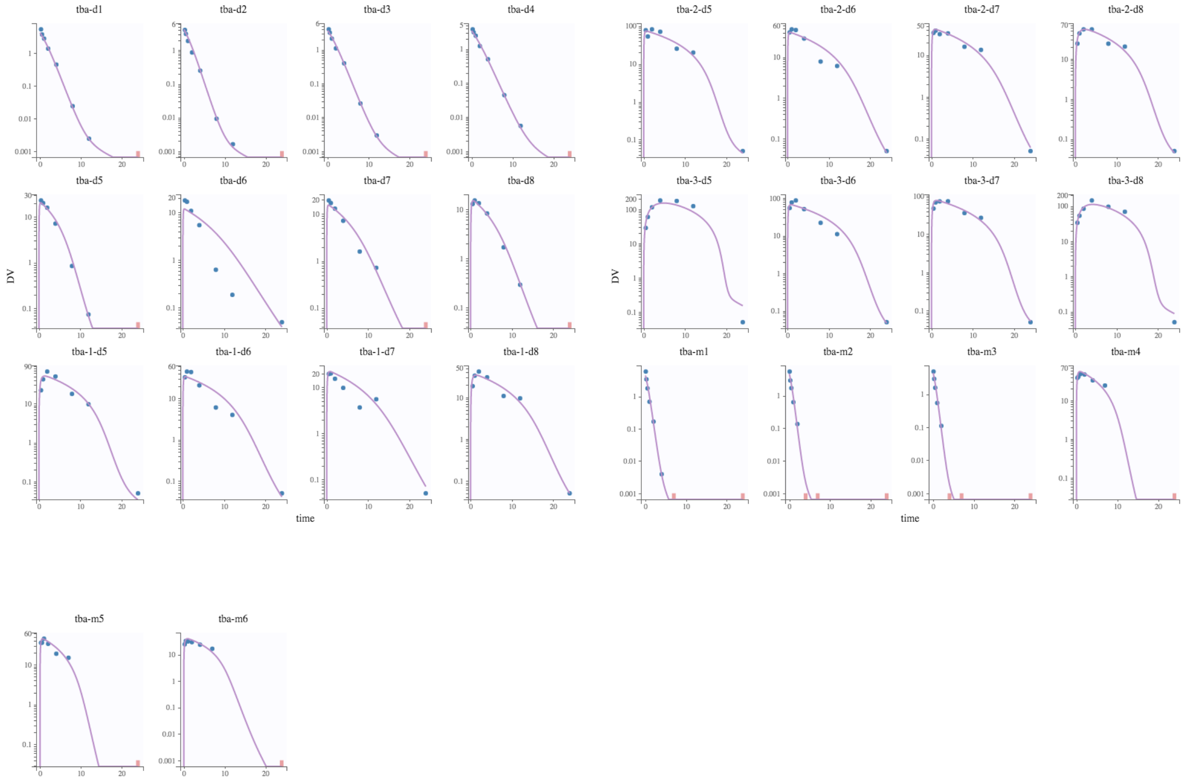

Figure S4: Model diagnostic check of individual fits for TBI-223

Appendix B: Clinical simulation code

```
[LONGITUDINAL]

input = {ka, V1, Q, V2, CLint, Km, Foral, kgrowth, kdeath, B0,

emax, ec50, gam, kp}


PK:

; Parameter transformations

V = V1

k12 = Q/V1

k21 = Q/V2

Vm = CLint*Km


; Plasma PK model definition

compartment(cmt=1, volume=V, concentration=Cc)


oral( cmt=1, ka, p = Foral)

peripheral(k12,k21)

elimination(cmt=1, Vm, Km)


EQUATION:

odeType = stiff




; Effect compartment concentration

if (Cc <= 0)

Cp = 0.000001
```

```
else
Cp = Cc
end

ddt_Ce = kp*(Cp-Ce) ;

Ce_c = Ce
;====== PD part of the model

Eff = (emax*Ce_c)/(ec50+Ce_c)

B_0 = B0 ; patient baseline CFU

;ODE for bacteria compartment
ddt_B = - B*Eff

[INDIVIDUAL]
input={ka_pop, omega_ka,
Cl_pop, omega_Cl,
V1_pop, omega_V1,
Q_pop, omega_Q,
V2_pop, omega_V2,
B0_pop, omega_B0}

DEFINITION:
;Omega
```

```
ka = {distribution = lognormal, prediction = ka_pop, sd = omega_ka}

CLint = {distribution = lognormal, prediction = Cl_pop, sd = omega_Cl}

V1 = {distribution = lognormal, prediction = V1_pop, sd= omega_V1}

Q = {distribution = lognormal, prediction = Q_pop, sd = omega_Q}

V2 = {distribution = lognormal, prediction = V2_pop, sd = omega_V2}

B0 = {distribution = lognormal, prediction = B0_pop, sd = omega_B0}
```
